# Preferences for postpartum pelvic floor muscle rehabilitation among pregnant women

**DOI:** 10.3389/fmed.2026.1766526

**Published:** 2026-03-11

**Authors:** Maodan Xu, Chaojian Tan, Hu Jiang

**Affiliations:** The Third Affiliated Hospital of Zunyi Medical University, The First People's Hospital of Zunyi, Zunyi, China

**Keywords:** DCE, patient preferences, patients, pelvic floor disorders, pregnancy

## Abstract

**Objectives:**

This study aimed to investigate the preferences of pregnant women for pelvic floor muscle rehabilitation following childbirth.

**Methods:**

A questionnaire-based survey was conducted from June 2025 to August 2025 using convenience sampling. Pregnant women attending the First People’s Hospital of Zunyi were recruited to rate their preferences for postpartum pelvic floor muscle rehabilitation treatments using a discrete choice experiment (DCE). The preferred attributes included rehabilitation techniques, rehabilitation costs, single-treatment duration, risk of side effects, rehabilitation locations, and operators. A mixed logit model was constructed using Python 3.11.7 software for preference analysis.

**Results:**

A total of 693 questionnaires were distributed, of which 670 valid responses were retrieved, yielding an effective response rate of 96.6%. The results indicated that rehabilitation techniques, rehabilitation costs, single-treatment duration, risk of side effects, rehabilitation settings, and service providers significantly influenced postpartum women’s preferences for pelvic floor muscle rehabilitation (*p* < 0.05). Among these factors, the risk of side effects was the most important determinant of pregnant women’s choice of pelvic floor muscle rehabilitation, followed by rehabilitation settings.

**Conclusion:**

Pregnant women prefer electrical stimulation and biofeedback therapy, with key preferences including costs under CNY ¥1,000, mild side effects, outpatient settings, qualified professionals, and 30-min sessions. To meet these needs, efforts should extend beyond individual departments to involve hospitals, health authorities, and community resources. The focus should be on reducing side effects and costs, enhancing treatment convenience, and developing personalized plans. This integrated approach will optimize resource allocation and improve access to effective rehabilitation services for postpartum women.

## Introduction

Pregnancy and childbirth are physiological processes that can induce varying degrees of damage to the female pelvic floor muscles, leading to a range of disorders such as urinary incontinence and pelvic organ prolapse. These conditions are relatively common among pregnant women and postpartum women (i.e., defined in most studies up to 6 weeks and in others up to 12 weeks after delivery) (1). A study indicates that within 6 to 8 weeks postpartum, the incidence of pelvic floor muscle injury exceeds 70%, urinary incontinence exceeds 40%, and pelvic organ prolapse exceeds 20% (1). These disorders not only compromise the physical health of pregnant and postpartum women but also exert adverse effects on their mental health, quality of life, and family relationships (1).

In recent years, significant progress has been made in pelvic floor muscle rehabilitation research targeting pregnant women and new mothers. Multiple rehabilitation methods, including bladder training, manual massage, pelvic floor muscle exercises, pelvic floor biofeedback therapy, and pelvic floor electrical stimulation, have been extensively studied and have been proven to yield clinical efficacy (2, 3). However, the successful implementation of postpartum pelvic floor muscle rehabilitation depends on active user participation and clinical integration. Despite clear rehabilitation benefits, the proportion of pregnant and postpartum women participating in pelvic floor muscle rehabilitation and their adherence remain suboptimal (4). For instance, while 51.8% of pregnant women expressed a desire to learn about pelvic floor muscle exercises and 56.0% planned to perform these exercises postpartum, only 11.3% maintained high exercise compliance (5). The absence or delay of rehabilitation therapy often leads to the persistence or worsening of pelvic floor dysfunction. Sustainable health-promoting behaviors can only be established when patients possess rehabilitation motivation and actively participate. While the majority of current studies focus on the implementation outcomes of rehabilitation interventions, there remains insufficient attention to the patient’s central role in the rehabilitation training process (6, 7).

With growing health awareness, the patient-centered shared decision-making model has become prominent in the field of obstetrics and gynecology, emphasizing information sharing between healthcare providers and patients to balance preferences (8). However, pregnant women’s preferences for pelvic floor muscle rehabilitation remain unclear, which significantly influences their engagement and outcomes (9). Factors such as health knowledge, cultural differences, access to healthcare, and individual experiences impact these preferences (10–12). Given the unclear preferences for pelvic floor muscle rehabilitation among pregnant women, the factors influencing these preferences are also unknown. This study aims to further clarify these factors.

The discrete choice experiment (DCE) is a valuable method for quantifying these preferences and is widely used in healthcare to analyze service preferences and inform economic evaluations and interventions (13). In pelvic floor rehabilitation, DCE helps explore pregnant and postpartum women’s preferences for methods, costs, and other attributes. However, DCE studies focusing on pregnant women remain limited, with existing research primarily covering prenatal care and perinatal mental health (14–16). The current research on the preferences of pregnant and postpartum women is insufficient, and further studies are needed to improve our understanding of their preferences.

To address these research gaps, this study aimed to analyze the key attributes and levels of pregnant women’s preferences regarding postpartum pelvic floor muscle rehabilitation. The findings are expected to provide evidence-based support for women to select appropriate treatment plans and for clinicians to make informed clinical decisions.

## Methods

### Design

This study was conducted using a cross-sectional survey design from June to August 2025. The subjects were pregnant women in Guizhou Province. We used convenience sampling to recruit participants. This method was chosen due to the accessibility and feasibility of reaching this specific population within the limited time frame of the study, ensuring a sufficient number of participants for preliminary analysis.

### Participants and setting

Data were collected at the Obstetrics Outpatient Department of the First People’s Hospital of Zunyi City. Participants met the following inclusion criteria: ① age ≥18 years; ② pregnancy status: entire pregnancy period (including early pregnancy <10 weeks, mid-pregnancy 11–20 weeks, late pregnancy 21–30 weeks, and beyond); ③ normal cognitive function with effective communication ability; and ④ voluntary participation and signed informed consent. The exclusion criteria included ① severe pregnancy complications or comorbidities impairing communication; ② history of mental illness; and ③ cognitive impairment, which was assessed through a brief cognitive function questionnaire designed to evaluate basic cognitive abilities such as memory, attention, and language comprehension. This ensured that participants could understand and respond appropriately to study instructions and consent forms.

### Sample size calculation

According to the “rule of thumb” proposed by Johnson and Orme, the minimum sample size required for a discrete choice experiment (DCE) is calculated using the formula: N > 500c/(t∗a) (17, 18), where, N represents the sample size, 500 is a constant, c denotes the maximum number of levels across all attributes, t is the number of choice sets per version, and a stands for the number of alternatives per choice set. In this study, we set c = 4, t = 10, and a = 3, yielding a minimum required sample size of 67. However, in consideration of real-world factors such as attrition rates and invalid questionnaires, the sample size should be expanded as much as possible to improve the accuracy and reliability of the research findings.

### Data collection

A uniformly trained researcher explained the purpose, methodology, and significance of the study to the participants, and the questionnaire was administered after obtaining informed consent. The researcher collected the contact information of the patients and their families in advance and scheduled an appointment with the patients. The patients filled out the questionnaires, which took approximately 15–20 min. The researcher promptly validated the data and excluded invalid questionnaires. At the end of the study, all data were double-checked and entered into the computer.

### Ethical considerations

This study was reviewed and approved by the Ethics Committee of the First People’s Hospital of Zunyi, with the assigned ethics approval number: (2025)-1–192. All participants provided written informed consent before enrolling in the study.

### Statistical analysis

Statistical analyses were performed using SPSS 26.0 and Python 3.11.7. A mixed logit regression model and a conditional logit regression model were established for preference analysis. The regression coefficients indicated the direction and magnitude of the influence of each attribute on the postpartum rehabilitation preferences of women during and after pregnancy. The Akaike Information Criterion (AIC) and Bayesian Information Criterion (BIC) were used to evaluate the goodness-of-fit of the two models, and the most appropriate regression model for this study was determined based on these results. According to regression model selection principles, smaller AIC and BIC values indicate a better model fit. Willingness to pay (WTP) reflects the monetary value that patients are willing to pay or accept as compensation when different attribute levels change. The significance level was set at *α* = 0.05.

## Measures

### Demographic questionnaire

The demographic questionnaire includes questions on the following demographic features: age, pregnancy weeks, childbirth history, number of existing children, educational level, occupational status, medical insurance, pelvic floor muscle rehabilitation status, personal monthly income, and pregnancy-related complications.

### Discrete choice experiment questionnaire design

The questionnaire development followed a rigorous process: First, a systematic literature review and semi-structured interviews identified potential dimensions and attributes related to maternal preferences for pelvic floor muscle rehabilitation. Second, the Delphi expert consultation method was used, involving two rounds of consultation with experts (*n* = 15) from obstetrics and gynecology, rehabilitation medicine, nursing, and health economics. Core attributes (e.g., rehabilitation model, location, cost, effectiveness, and time commitment) and their specific levels were selected and finalized based on importance scores, coefficient of variation, and expert opinions. Finally, based on the selected attributes and levels, a discrete choice experiment (DCE) questionnaire version was designed using Ngene software. A small-scale pre-survey (*n* = 30) tested the questionnaire’s comprehensibility, reliability, and validity, enabling further refinement of wording and option settings.

This study developed a situational questionnaire based on women’s preferences for postpartum rehabilitation services. Using the Comprehensive Framework for Implementation Research (CFIR), potential attributes were identified through a literature review and key informant interviews with experts. Six attributes of multiple levels were selected (Table 1). To manage complexity, an efficient design method using D-efficiency criteria generated 24 experimental scenarios, which were divided into 8 multiple-choice questions with three options each, including an “opt-out” option. A “consistency check question” was added to validate responses.

**Table 1:** Attributes and their levels in the discrete-choice experiment.

Attributes	Attributes levels	Explanation
Rehabilitation methods	① Sports rehabilitation ② Electrical stimulation therapy ③ Laser/radiofrequency therapy ④ Biofeedback	A standardized set of medically assessed and personalized training methods designed to promote recovery after pelvic floor muscle injury
Rehabilitation expenses	① Free (covered by medical insurance) ② <CNY ¥1,000 ③ CNY ¥1,000–3,000 ④ >CNY ¥3,000	The full expense for services such as pelvic floor assessment and training, as determined by medical standards
Single treatment duration	① 30 min ② 1 h ③ 1.5 h	The standard recommended length of one pelvic floor rehabilitation session
Side effect risk	① Risk-free ② Mild discomfort (such as pain) ③ Moderate risk (such as infection) ④ High-risk (surgical complications)	Potential adverse effects that may occur during pelvic floor rehabilitation due to the nature of the procedure or individual factors
Rehabilitation facility	① Home ② Hospital outpatient department ③ Maternity Center ④ Community	A qualified medical institution that provides professional pelvic floor assessment and rehabilitation services
Operator	① Doctor ② Nurse ③ Operate independently ④ Other	A trained professional who performs pelvic floor assessment and rehabilitation

CNY, Chinese Yuan.

## Results

### Demographics

A total of 693 questionnaires were distributed, and after excluding 23 invalid responses, a 96.6% response rate was achieved, resulting in 670 valid questionnaires from pregnant women. The majority of participants were aged 31–35 years (34.33%). In terms of gestational age, 30.75% were between 21 and 30 weeks. The majority of participants were primiparous (74.83%) and had one child (58.06%). Regarding education, 39.70% held a university degree or higher. Employed women accounted for 31.94% of the sample, and 45.82% were covered by employee medical insurance. The details are presented in Table 2.

**Table 2:** Basic information of participants (*n* = 670).

Variables	Number	Percentage (%)
Age (year)		
<25	137	20.45
25–30	172	25.67
31–35	230	34.33
≥36	131	19.55
Pregnancy weeks		
≤10	114	17.01
11–20	132	19.70
21–30	206	30.70
≥31	218	32.54
Childbirth history		
First-time mother	168	25.07
Second-time mother	502	74.93
Number of children		
0	168	25.07
1	389	58.06
2	107	15.97
3	6	0.90
Educational level		
Junior high school and below	110	16.42
High school / Technical secondary school	218	32.54
University or above	342	51.04
Occupational status		
Employed	214	31.94
Full-time maternity	229	34.18
Freelance work	175	26.12
Other	52	7.76
Medical insurance		
Employee medical insurance	307	45.82
Resident medical insurance	340	50.75
Other	23	3.43
Experience of pelvic floor muscle rehabilitation		
Yes	274	40.90
No	396	59.10
Personal monthly income		
< CNY ¥3,000	118	17.61
CNY ¥3,000–6,000	195	29.10
CNY ¥6,001–9,000	194	28.96
≥CNY ¥9,001	163	24.33
Pregnancy complications		
Yes	227	33.88
No	443	66.12

CNY, Chinese Yuan.

### Discrete choice experiment results

The data were analyzed using both a conditional logit model and a mixed logit model. The Akaike Information Criterion (AIC) and Bayesian Information Criterion (BIC) values for the conditional logit model were 13,555.3 and 13,690.85, respectively, while those for the mixed logit model were 13,543.58 and 13,695.06. Given that a lower AIC suggests a better balance between model fit and simplicity, the mixed logit model was considered more suitable. This study thus reports the results of the mixed logit model regression.

The analysis indicated that several attributes significantly influenced preferences for postpartum rehabilitation among women during and after pregnancy (all *p* < 0.05). These attributes include rehabilitation methods, cost, single treatment duration, side effect risks, rehabilitation location, and operator. Specifically, women were more inclined to prefer electrical stimulation therapy and biofeedback as rehabilitation methods (*β* = 0.211 and *β* = 0.268, respectively), with a cost of ≤CNY ¥1,000 (*β* = 0.170), mild discomfort as the side effect risk (*β* = 0.333), hospital outpatient departments as the rehabilitation location (*β* = 0.315), and “other” operators (β = 0.266). Additionally, a 30-min single treatment session was the most preferred duration (*β* = −0.347 for 1 h and *β* = −0.174 for 1.5 h, with 30 min as the reference level). These preferences are detailed in Table 3.

**Table 3:** Analysis results of preferences for pelvic floor muscle rehabilitation among pregnant and postpartum women.

Attributes and Levels	*β*	*SE*	*z*	*p-*value	*95%CI*
Rehabilitation methods					
Sports rehabilitation (reference)					
Electrical stimulation therapy	0.211	0.073	2.890	0.004	0.068 ~ 0.355
Laser/radiofrequency therapy	−0.486	0.063	−7.750	0.000	−0.609 ~ −0.363
Biofeedback	0.268	0.057	4.710	0.000	0.156 ~ 0.379
Cost					
Free (reference)					
≤CNY ¥1,000	0.170	0.046	3.700	0.000	0.080 ~ 0.261
CNY ¥1,000–3,000	−0.316	0.052	−6.020	0.000	−0.418 ~ −0.213
≥CNY ¥3,000	−0.387	0.048	−7.990	0.000	−0.482 ~ −0.292
Side effect risks					
No risk (reference)					
Mild discomfort	0.333	0.043	7.790	0.000	0.249 ~ 0.416
Moderate risk	−0.275	0.044	−6.300	0.000	−0.361 ~ −0.190
High risk	−0.051	0.041	−1.250	0.212	−0.130 ~ 0.029
Rehabilitation location					
Home (reference)					
Hospital outpatient department	0.315	0.049	6.460	0.000	0.219 ~ 0.411
Maternity center	0.018	0.050	0.370	0.713	−0.079 ~ 0.116
Community	0.053	0.050	1.040	0.298	−0.046 ~ 0.151
Operator					
Doctor (reference)					
Nurse	−0.098	0.046	−2.130	0.033	−0.188 ~ −0.008
Self-operation	0.096	0.061	1.570	0.116	−0.024 ~ 0.215
Other	0.266	0.039	6.820	0.000	0.190 ~ 0.343
Single treatment duration					
30 min (reference)					
1 h	−0.347	0.107	−3.230	0.001	−0.557 ~ −0.137
One and a half hours	−0.174	0.098	−1.780	0.076	−0.366 ~ 0.018

*β*, beta coefficient; SE, standard error; *z*, *z*-statistic; *p*-value, probability value; 95%CI, 95% confidence interval; CNY, Chinese Yuan.

### WTP analysis

According to the willingness-to-pay (WTP) analysis, pregnant women expressed distinct preferences across various rehabilitation attributes. For rehabilitation methods, compared to sports rehabilitation, they showed the highest WTP for biofeedback therapy (CNY ¥692.5), followed by electrical stimulation therapy (CNY ¥545.2), while demonstrating strong resistance to laser/radiofrequency therapy with a negative WTP of - CNY ¥1255.8. In terms of cost, relative to free rehabilitation, women were willing to pay only CNY ¥0.170 to keep the cost within CNY ¥1,000; WTP turned negative when costs increased to CNY ¥1,000–3,000 (−0.316 CNY) or above CNY ¥3,000 (− CNY ¥0.387). Regarding side effect risks, they were willing to pay CNY ¥860.5 to reduce the risk of mild discomfort to none; WTP fell to - CNY ¥710.6 for moderate risk, and was - CNY ¥131.8 for high risk, though the latter was not statistically significant (*p* = 0.212). For rehabilitation location, compared to home-based rehabilitation, women were willing to pay CNY ¥814 for hospital outpatient services, while WTP for maternity centers (CNY ¥46.5) and community settings (CNY ¥137) was not significant. Concerning the operator, compared to a doctor, WTP for other operators was CNY ¥687.3, whereas it was -CNY ¥253.2 for a nurse and CNY ¥248.1 for self-operation, though the latter was not significant. Finally, for single treatment duration, extending from 30 min to 1 h was associated with a WTP of - CNY ¥896.6, and extending to 1.5 h with -CNY ¥449.6 (*p* = 0.076). Detailed results are provided in Table 4.

**Table 4:** Willingness to pay for each attribute and level.

Attributes and levels	*WTP* (CNY)	SD	*p-*value	95%*CI*
Rehabilitation methods				
Sports rehabilitation (reference)				
Electrical stimulation therapy	545.2	0.073	0.004	175.700 ~ 917.300
Laser/radiofrequency therapy	−1255.8	0.063	0.000	−1573.600 ~ −938.000
Biofeedback	692.5	0.057	0.000	403.100 ~ 979.300
Cost				
Free (reference)				
≤CNY ¥1,000	0.170	0.046	0.000	0.080 ~ 0.261
CNY ¥1,000–3,000	−0.316	0.052	0.000	−0.418 ~ −0.213
≥CNY ¥3,000	−0.387	0.048	0.000	−0.482 ~ −0.292
Side effect risks				
No risk (reference)				
Mild discomfort	860.5	0.043	0.000	643.400 ~ 1074.900
Moderate risk	−710.6	0.044	0.000	−932.800 ~ −491.000
High risk	−131.8	0.041	0.212	−335.900 ~ 74.900
Rehabilitation location				
Home (reference)				
Hospital outpatient department	814	0.049	0.000	565.900 ~ 1062.000
Maternity center	46.5	0.050	0.713	−204.100 ~ 299.700
Community	137	0.050	0.298	−118.900 ~ 390.200
Operator				
Doctor (reference)				
Nurse	−253.2	0.046	0.033	−485.800 ~ −20.700
Self-operation	248.1	0.061	0.116	−62.000 ~ 555.600
Other	687.3	0.039	0.000	491.000 ~ 886.300
Single treatment duration				
30 min (reference)				
1 h	−896.6	0.107	0.001	−1439.300 ~ −354.000
1.5 h	−449.6	0.098	0.076	−945.700 ~ 46.500

WTP, willingness to pay; SD, standard deviation; *p*-value, probability value; 95%CI, 95% confidence.

## Discussion

This study used a discrete choice experiment to systematically investigate pregnant women’s preferences for pelvic floor muscle rehabilitation. Initially, we conducted literature reviews, consulted experts, and interviewed the target population. Based on these efforts, we established a preliminary attribute pool covering multiple dimensions, such as rehabilitation model, cost, efficacy, duration, and location. Through two rounds of Delphi expert deliberation, six core attributes were ultimately selected and confirmed based on importance, coefficient of variation, and clinical feasibility: rehabilitation model, cost, single-session duration, risk of side effects, implementation location, and provider. Analysis indicates that all of these attributes significantly influence maternal rehabilitation choice decisions.

The selection of rehabilitation methods significantly influences outcomes for pregnant women. Among available options, electrical stimulation therapy and biofeedback are frequently preferred due to their operational simplicity and established efficacy (19–21). As a mature technique, electrical stimulation therapy passively engages the pelvic floor muscles, making it particularly suitable for early postpartum women with weak muscle strength and difficulty initiating active contractions (22). Biofeedback, through intuitive signal feedback, assists pregnant and postpartum women in mastering correct pelvic floor muscle contraction, thereby enhancing the initiative and accuracy of rehabilitation (23–25). In contrast, while exercise rehabilitation is effective, it demands greater patient motivation and movement standardization. Without professional guidance, many women struggle to maintain consistency or perform exercises correctly, limiting training outcomes (26). Research indicates that difficulty in recalling prescribed movements often leads to poor or insignificant short-term results in exercise-based approaches. Consequently, biofeedback tends to be favored among this population (27).

Rehabilitation costs significantly influence pregnant women’s choices, with many preferring plans under CNY ¥1,000. In areas with uneven economic development, financial constraints often cause women to delay or skip pelvic floor muscle rehabilitation due to inadequate medical insurance and low reimbursement (28). Group-based rehabilitation and early initiation during pregnancy can also reduce costs. Group training for eight pregnant women saves $1,400, and it is more than 60% cheaper than individual treatment (29). Innovating service models can thus lower costs and enhance rehabilitation accessibility for these women. The duration of rehabilitation treatments and the role of service providers significantly influence the choices of pregnant and postpartum women. These women prefer shorter treatment durations, such as 30 min, due to the time constraints they face from childcare and family responsibilities (30). Longer treatments reduce their willingness to pay and compliance with rehabilitation. Studies show that time scarcity is a key barrier to persisting with pelvic floor muscle exercises, while having sufficient time is a facilitator (30). In clinical practice, cumbersome and prolonged rehabilitation programs often lead to high dropout rates among postpartum women (31).

The findings of this study indicate that the risk of side effects is the most influential factor in the decision-making process when pregnant women and new mothers select pelvic floor muscle rehabilitation programs. This highlights a pronounced tendency among this group to prioritize safety in their healthcare choices. These findings align with previous research that has reported varying degrees of adverse reactions during pelvic floor muscle training and biofeedback-based interventions (24, 32). The safety of medical interventions is a fundamental aspect of patient decision-making. High side effect risks during rehabilitation can impact outcomes and increase psychological stress, potentially leading to resistance against treatment (33, 34). To improve acceptance and adherence to pelvic floor muscle rehabilitation, it is imperative for healthcare providers to focus on minimizing side effect risks. It is advised that medical staff rigorously evaluate rehabilitation plans, favoring methods with a proven clinical track record and low side effect risks, and enhance monitoring to promptly address any discomfort. Furthermore, comprehensive disclosure of side effect risks and management strategies associated with different rehabilitation methods to these women can facilitate informed decision-making and bolster trust in the treatment process.

This study found that, when choosing pelvic floor muscle rehabilitation, pregnant women consider the rehabilitation venue to be the second most important factor. These women strongly prefer hospital outpatient clinics, even to the point of being willing to pay an extra CNY ¥814 to switch from home-based rehabilitation to hospital outpatient services. The preference is primarily due to the better professional resources available in hospital outpatient departments. These clinics have specialized rehabilitation equipment and qualified practitioners who can provide more standardized and professional treatment. This effectively meets the high standards that pregnant and postpartum women have for rehabilitation outcomes and safety. Empirical evidence also supports this advantage. A comparative study between outpatient and home-based pelvic floor muscle exercises showed that 41% of home-based participants needed continued physical therapy after 1 year because their results were not satisfactory (35, 36). Similarly, the cure rate for severe urinary incontinence was much higher among outpatient recipients (62%) compared to home-based patients (28%) (37). While home-based rehabilitation is convenient, its effectiveness is limited by the lack of professional supervision and support (38). This aligns with evidence that supervised pelvic floor muscle exercises are more beneficial for pregnant women (39).

This study indicates that pregnant and postpartum women prioritize safety, professionalism, and affordability when selecting rehabilitation services. Regarding the specific attribute of the operator, professional identity significantly influences their willingness to pay (WTP): using physicians as the reference, WTP was the highest; WTP for services provided by nurses was significantly lower, which may stem from insufficient recognition of their specialized rehabilitation skills; while the “other” category (e.g., rehabilitation therapists) demonstrated clear demand. This suggests that service design should move beyond the general notion of “having a professional available” and instead clearly articulate the distinct professional value of different roles. To systematically address these preferences, clinical pathways should integrate safety protocols and optimize communication, while at the service delivery level, efforts should explore insurance coverage, appointment optimization, and hybrid outpatient-home models, ultimately enhancing the accessibility, safety, and effectiveness of services.

### Limitations

This study has the following limitations. First, the sample originates from a specific region, potentially introducing regional bias and limiting the generalizability of findings to broader populations. Second, data are based on participants’ self-reported preferences, which may differ from actual choice behaviors. Third, the absence of long-term follow-up data prevents the assessment of sustained adherence to rehabilitation programs and their long-term health outcomes. As some authors have noted in the literature, long-term follow-up is essential not only to achieve good outcomes but also to ensure therapeutic adherence. Future studies should consider incorporating additional attributes, such as the preferred type of follow-up (e.g., telephone or in-person) and follow-up duration, to better capture participants’ follow-up preferences. Additionally, some participants had no prior experience with pelvic floor muscle rehabilitation, which means their stated preferences may reflect expectations or theoretical perceptions rather than preferences based on actual experience. This could potentially affect the validity of their responses and should be considered when interpreting the results.

## Conclusion

This study indicates that Chinese pregnant women prioritize safety, convenience, professionalism, and technological efficacy in pelvic floor muscle rehabilitation, while also demonstrating cost sensitivity, with most accepting costs within CNY ¥1,000. These preferences highlight a demand for effective, accessible, and affordable care. However, the study’s conclusions are limited to pregnant women and do not address the postpartum period. Future research should extend the analysis to include postpartum women to provide a more comprehensive understanding. To enhance service uptake, rehabilitation programs should focus on mitigating risks and costs, strengthening professional capacity, and offering evidence-based, personalized plans. Additionally, further exploration of attributes and levels beyond cost is recommended to fully address the study’s objective.

## Data Availability

The raw data supporting the conclusions of this article will be made available by the authors, without undue reservation.
